# An Exploratory Study on the Effectiveness of Virtual Reality Analgesia for Children and Adolescents with Kidney Diseases Undergoing Venipuncture

**DOI:** 10.3390/ijerph19042291

**Published:** 2022-02-17

**Authors:** Barbara Atzori, Laura Vagnoli, Daniela Graziani, Hunter G. Hoffman, Mariana Sampaio, Wadee Alhalabi, Andrea Messeri, Rosapia Lauro-Grotto

**Affiliations:** 1 Department of Health Sciences, University of Florence, 50134 Florence, Italy; rosapia.laurogrotto@unifi.it; 2 Pediatric Psychology Service, Meyer Children’s Hospital, 50139 Florence, Italy; laura.vagnoli@meyer.it (L.V.); danielagraziani@yahoo.it (D.G.); 3 Department of Mechanical Engineering HPL, University of Washington, Seattle, WA 98195, USA; hunthoff9@gmail.com; 4 Department of Computer Science, King Abdulaziz University, Jeddah 21589, Saudi Arabia; wsalhalabi@kau.edu.sa; 5 Department of Psychology, University of Coimbra, 3000-115 Coimbra, Portugal; marimakai@gmail.com; 6 Department of Social Work, Catholic University of Portugal, 1649-023 Lisbon, Portugal; 7 Immersive Virtual Reality Research Group, King Abdulaziz University, Jeddah 21589, Saudi Arabia; 8 Department of Computer Science, Dar Alhekma University, Jeddah 21589, Saudi Arabia; 9 Pain Therapy and Palliative Care, Meyer Children’s Hospital, 50139 Florence, Italy; andrea.messeri@uslcentro.toscana.it; 10 Laboratory for Multidisciplinary Analysis of Relationship in Health Care, 51100 Pistoia, Italy

**Keywords:** virtual reality, kidney disease, pain, venipuncture, children health

## Abstract

The current study evaluated the effectiveness of VR analgesia among pediatric and adolescent patients with kidney disease undergoing venipuncture. Patients at an Italian Children’s hospital (N = 82, age range 7–17 years) undergoing venipuncture were randomly assigned to a No VR group (non-medical conversation) vs. a Yes VR group (VR analgesia). After the procedure, patients gave 0–10 Verbal Numeric Pain Scale ratings. Compared with patients in the No VR Group, patients in the Yes VR group reported significantly lower “Pain intensity”(No VR mean = 2.74, SD = 2.76 vs. Yes VR mean = 1.56, SD = 1.83) and the VR group also rated “Pain unpleasantness” significantly lower than the No VR group (No VR mean = 2.41, SD = 2.94 vs. Yes VR mean = 1.17, SD = 1.80). Patients distracted with VR also reported having significantly more fun during the venipuncture procedure. No side effects emerged. In addition to reducing pain intensity, VR has the potential to make venipuncture a more fun and less unpleasant experience for children with CKD, as measured in the present study for the first time. Finally, in exploratory analyses, children aged 7–11 in the VR group reported 55% lower worst pain than control subjects in the same age range, whereas children aged 12 to 17 in the VR group only reported 35% lower worst pain than control subjects. Additional research and development using more immersive VR is recommended.

## 1. Introduction

Pediatric pain management in hospitals is a priority [1,2]. There is strong evidence that undertreated pain has damaging effects on patient’s experience during medical procedures, and excessive procedural pain is predictive of higher levels of pain and anxiety during the successive procedures [3,4,5]. Furthermore, unpleasant medical experiences can lead to avoidance of hospitals, reducing preventative medicine, and increasing healthcare costs [6]. As analyzing blood has become increasingly sophisticated, needle-related procedures are some of the most common painful procedures carried out in hospitals. Venipuncture or blood draws are especially common, and can often be experienced as unpleasant, generating anxiety and avoidance, especially in children [7,8]. Fear of needles is a common phobia that can trigger fight or flight responses from the patient before and during the procedure, and can make patients less cooperative. In a study of young children by Lunoe et al., 67% of patients had high anxiety before their vaccination, while moderate to severe pain during vaccination was observed in 65% of the children [9]. In a related study of children aged 7–18 years old, Hedén et al. (2019) found that children’s fear level during needle insertion was positively correlated with their pain level. In other words, higher fear levels predicted higher pain during venipuncture [10]. A meta-analysis by McLenon and Rogers found that fear of needles is very common, e.g., the majority of children in the studies analyzed exhibited fear of needles, while in adults, according to their surveys, 27% of hospital employees avoided flu shots because of fear of needles [11]. Lunoe et al. recommended anxiety reducing interventions to help reduce pain during venipuncture [9].

Why are most children afraid of needles when venipuncture often lasts only a few seconds or a few minutes at most? Pain intensity can be strongly influenced by psychological factors, which can amplify how much pain patients subjectively experience. Anticipation that a venipuncture is going to be painful can amplify the nociceptive signal, increasing pain-related brain activity [12]. Fear can be a learned behavior. For example, if a child who is about to get a blood draw sees that their parent is afraid of needles, this can make the procedure more painful/anxiety provoking for the child. Watching (focusing their attention on) the medical procedure/wound care can also increase how much pain patients experience during painful medical procedures [13]. One study found that pain catastrophizing (exaggerated fears about pain) is correlated with venipuncture pain [14], and unpleasant memories for previous painful medical experiences can influence subsequent experiences [15]. Fortunately, non-drug psychological treatments such as distraction can help reduce pain [16]. Distraction can reduce ruminating anticipation of pain and can reduce pain by simply getting the patient to think about something unrelated to their medical procedure. Distraction is often used in addition to traditional pain medications.

Virtual Reality (VR) is emerging as a promising distraction technique for children’s pain management [17]. Immersive virtual reality (VR) has been found to reduce pain, anxiety, and distress both in adults and children during a growing number of painful medical procedures [18]. Several previous studies have explored the use of virtual reality to distract children during venipuncture, with mixed results. In a small early study by Gold and Kim et al., children with unspecified medical conditions received an IV placement before MRI/CT scans. VR did not significantly reduce pediatric patients’ ratings of pain intensity during venipuncture [19]. Similarly, Dumoulin et al. found that VR significantly reduced fear and increased patient satisfaction, but did not significantly reduce pain during venipuncture [20]. In a pilot study, Atzori and Hoffman et al. found that VR significantly reduced pain during venipuncture in children and adolescents with cancer [17]. Similarly, Özalp Gerçeker et al. found that VR reduced pain, fear, and anxiety in children aged 5–12 during blood draws [21]. In a recent Randomized Controlled Trial study of children, adolescents, and young adults with unspecified diseases, VR significantly reduced pain intensity [22]. Similarly, in their recent study of VR distraction during peripheral intravenous catheter placement in children of unspecified diseases, Gold et al. found significant reductions in pain intensity when using VR [23].

For children suffering from chronic diseases, such as chronic kidney diseases (CKD), venipuncture is an essential procedure periodically needed for checking their medical condition. Chronic kidney disease can range from mild to end-stage renal disease, requiring dialysis or transplantation because of kidney failures [24,25], and CDK is associated with higher rates of hospitalization [26] and disability [27]. CKD can emerge early during childhood, because of congenital kidney and urinary anomalies (hypoplasia, dysplasia, or urinary tract obstruction), or it can be associated with different conditions such as prematurity, obesity, diabetes, and cardiovascular disorders. To avoid the disease progression and the related consequences in pediatric patients with CKD, persistent health status monitoring and early treatments are essential [28]. The current study is one of the first to evaluate the effectiveness of VR analgesia among pediatric and adolescent patients with kidney disease undergoing venipuncture. Based on the logic proposed by Hoffman et al. [29], we predicted that VR would draw patients’ attention into the VR world, leaving less attention available to process incoming pain-related information, with the result of a reduced pain perception during venipuncture. To date, we are aware of only one previous venipuncture study of children and adolescents with our specific patient population, i.e., children with chronic kidney disease. Piskorz and Czub reported significantly lower levels of pain intensity and stress for the group that received VR [30]. The current study attempted to replicate and extend Piskorz and Czub’s [30] findings that VR reduces pain intensity during pediatric venipuncture in CKD. In addition to measuring the sensory component of pain (worst pain intensity), the current study innovatively measured, for the first time in CDK patients, the emotional component of pain during venipuncture (pain unpleasantness), the cognitive component of pain (time spent thinking about pain), and fun during venipuncture, a surrogate measure of positive emotion in CDK patients. In addition to reducing pain intensity, VR has the potential to make venipuncture a more fun and less unpleasant experience for children with CKD, as measured in the present study for the first time. The current study also reported exploratory descriptive analyses (% reduction in pain during VR) in patients aged 7–11 vs. 12–17 year olds.

## 2. Materials and Methods

### 2.1. Participants

The current study was conducted in the Service of Nephrology and Dialysis at a Children’s hospital in Italy. From November 2017 to April 2018, patients undergoing venipuncture for their periodic blood analysis were recruited. Patients aged 7–17 years with different kidney diseases and who were able to understand Italian language and to complete the tests were selected with the help of a nurse. According to the exclusion criteria adopted in previous VR studies, we excluded children who were unable to wear the helmet and interact with the VR environment, with a diagnosis of epilepsy, with physical or psychological impairments, and participants not accompanied by their legal caregivers [17]. Moreover, patients who had never undergone blood analysis before or new patients without a definite diagnosis of kidney disease were excluded from the study.

### 2.2. Procedure

The protocol was accepted by the ethical committee of the hospital. The study was approved by doctors and nurses of the Service of Nephrology and Dialysis and conducted in collaboration with the Service of Pediatric Psychology and the Service of Pain Therapy and Palliative Care. Patients meeting the inclusion criteria and their families were approached by the psychologist researcher in the waiting room in order to determinate their interest. Interested patients and their parents (or legal guardians) were accompanied by the psychologist researcher into the room of the procedure to complete the written informed assent/consent forms. Using computer-generated random-number sequences obtained from a statistician not involved in data collection, patients were randomly assigned to either the No VR group or the VR group. For both the No VR and the VR group, the parent/legal guardian remained in the room for the entire medical procedure. The No VR control group (standard treatment as usual) consisted of non-medical conversation by the nurse who performed venipuncture with the patient. In the Yes VR group (experimental group), patients interacted with VR during venipuncture. Before the beginning of the procedure, patients in the VR group received 5 min of instructions to learn how to wear the helmet and to interact with the VR environment, with the help of the psychologist researcher. The helmet and the earphones included in the VR system were worn immediately before the beginning of the venipuncture and removed immediately after the end of the blood draw. In both groups the nurse remained in the room all the time as usual.

### 2.3. Measures

Worst pain, the primary outcome measure, was assessed at the end of the venipuncture, using a 0–10 Verbal Numeric Rating Scale. Participants were asked to rate their worst pain during the venipuncture (0 = “No pain” to 10 = “Excruciating pain”). Verbal Numeric Rating Scales have been validated for use in children aged 6 and higher [31]. According to [31], Verbal Numeric Rating Scales have strong convergent validity, known-groups validity, responsivity, and reliability for children aged 6 to 17 years. In secondary measures also using Verbal Numeric Rating Scales, the affective component was investigated, asking how unpleasant the venipuncture was (0 = “Not at all unpleasant” to 10 = “The most unpleasant”) [32,33]. The cognitive component of pain was investigated by asking patients how much time they spent thinking about their pain during the procedure (0 = “None” to 10 = “All the time”). All patients were also asked how much “fun” they had during the procedure (0 = no fun at all, 10 *=* extremely fun; Hoffman, Sharar, Coda et al., [34]), and their nausea levels (0 = “None” to 10 = “Vomit”). Only patients in the Yes VR group also rated the quality of their VR experience with a brief structured interview and also gave a score between 0–10 on a Verbal Numeric Pain Scale to the following two questions: (1) While experiencing VR, to what extent did you feel like you went into the virtual world? (0 = “I did not feel like I went inside at all” to 10 = “I went completely inside the computer-generated world”, Hoffman et al. [29], adapted from Slater et al., [35]); and patients also rated (2) How real did the objects in the virtual world seem to you? (0 = “Completely fake” to 10 = “Indistinguishable from real objects”, [29]).

### 2.4. Immersive Virtual Reality System

**Figure 1:**
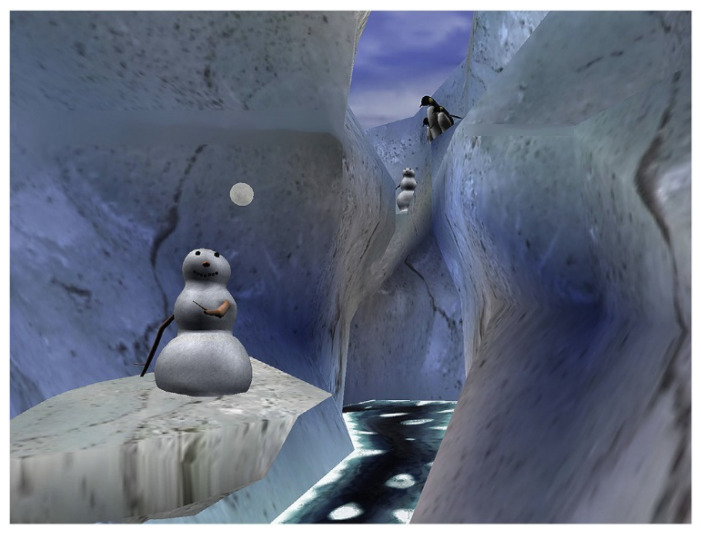
In SnowWorld, patients “go into” an icy canyon, where they throw snowballs at penguins, snowmen and other characters in VR. Image by Ari Hollander and Howard Rose, copyright Hunter Hoffman, www.vrpain.com accessed on 5 December 2021.

The VR equipment consisted of a VR helmet, the Personal 3D Viewer Sony: HMZ T-2, supported by a laptop, which allowed the interaction with the VR environment. The helmet had a 45° diagonal field of view, 1280 × 720 pixels per eye, and was suitable for younger patients. The VR helmet had two miniature screens, one for each of the user’s eyes, and earphones to provide acoustic isolation and increase presence in VR, the illusion of “being there” in the computer-generated world as if it is a place they are visiting. The VR software used was called SnowWorld (see Figure 1), one of the most frequently employed virtual reality environments specifically designed to promote distraction from procedural pain [17]. In SnowWorld, patients “go into” an icy canyon, where they float slowly through the canyon while throwing snowballs at penguins, snowmen, and other characters in VR, using a wireless mouse with the hand not employed in the venipuncture.

### 2.5. Data Analysis

Descriptive statistics were used to characterize the study sample and to describe the % reduction in worst pain in children aged 7–11 vs. 12–17; *t*-tests and chi-squared analyses were used to compare demographic variables in the two groups. A *t*-test for independent samples was used to compare pain, fun, and nausea between the No VR group and the Yes VR group. A researcher not involved in data collection generated the random-number sequences used for random assignment to groups, and carried out data analysis using the statistical Software SPSS 25 (IBM Corp, Armonk, NY, USA). Results were considered significant when associated with *p* values less than 0.05.

## 3. Results

### 3.1. Sample

Eighty-two patients (46.3% females, 53.7% males, mean age 11.78 years, SD = 2.70) took part in the study and were randomly assigned to the control or experimental group. None of the patients had previously used a VR system, except two males in the experimental group: one had an HTC VIVE VR system at home and the other one had played a videogame with a pair of Sony VR goggles in an arcade. Sample demographics variables are reported in Table 1.

### 3.2. Pain

As shown in Table 2, patients distracted by VR reported significantly lower scores for the sensory (worst pain) and emotional (pain unpleasantness) components of pain. “*Worst pain*”: No VR group mean 2.74, SD = 2.76 vs. Yes VR group mean 1.56, SD = 1.83; *t*(80) = 2.29, *p* < 0.05), and “*Pain unpleasantness*”: No VR group mean 2.41, SD = 2.9 vs. Yes VR group mean 1.17, SD = 1.80; *t*(80) = 2.31, *p* < 0.05. The predicted pattern of higher mean levels of the cognitive component of pain (“Time spent thinking about pain”) was reported by patients in the No VR group vs. Yes VR group. However, the difference was not significant (*p* > 0.05) for this variable. 

### 3.3. Fun and Nausea (on A Scale from Zero to 10)

A significant difference for fun levels emerged between the two groups: No VR group mean 4.06 (SD = 3.74), vs. Yes VR group mean = 8.06 (SD = 1.85), *t*(80) = −6.14 (*p* < 0.001). No significant differences emerged for nausea levels between the two groups (*p* > 0.05): in both groups, patients reported mean levels of nausea less than 1 (on a 0 to 10 scale).

### 3.4. VR Experience

On a scale from 0 to 10, where 10 = “I went completely into the computer generated world”, patients who interacted with VR reported a mean presence score of 7.11 (SD = 3.00). On a scale from 0 to 10 where 10 = “indistinguishable from a real object”, mean realism of VR objects was 6.05 (SD = 3.12). Seventy-one percent of patients reported presence levels higher than 5 (on a scale from 0 to 10) and the 29% of the patients reported presence levels lower or equal to 5. Sixty three percent of patients also reported a score higher than 5 (on a scale from 0 to 10) when asked “How real did the objects in the virtual world seem to you”.

In post-hoc exploratory analyses (see Table 3), patients aged 7–11 (younger children) reported a 55% reduction in worst pain during VR and as shown in Table 4, patients aged 12–17 (older children) reported a 35% reduction in worst pain.

## 4. Discussion

The current study aimed to evaluate VR as a distraction technique for pain management in children with chronic kidney disease undergoing venipuncture. As predicted, children and adolescent patients with kidney disease reported a significant reduction in pain intensity (worst pain) when they used VR compared with the No VR group. Results showed for the first time in CKD patients that in addition to reducing sensory (i.e., worst) pain, VR distraction was also useful for managing the emotional component of pain as indicated by the significantly lower levels of pain unpleasantness and significantly more fun during venipuncture reported by patients in the Yes VR group compared with the patients in the No VR control group. Patients who used VR also reported the predicted pattern of lower “Time spent thinking about pain” levels, however the difference between the control and the experimental group was not significant for this variable.

The results of our primary outcome measure (worst pain) are in line with the recent results of Piskorz and Czub’s study, where pediatric patients of a nephrology clinic undergoing venipuncture and distracted with VR reported significantly lower levels of pain and stress compared with patients in the No VR control group [30]. The current results are innovative in that this is the first CKD study to measure the effects of VR on the emotional and cognitive components of pain, and, importantly, this study is the first to show that CKD patients who received VR had significantly more fun during venipuncture than the No VR control group, presumably making the venipuncture a more positive experience for patients who received VR. Using exploratory analyses, this is also the first CKD VR study to describe the percentage of VR analgesia pain reduction in patients aged 7–11 vs. 12–17. Children aged 7–11 reported 55% less pain intensity during VR, whereas children aged 12–17 reported a 35% reduction in pain intensity compared to age-matched patients in the control group.

### 4.1. The Mechanism of How VR Reduces Acute Pain

Beginning with the first study on immersive VR analgesia [29,36], a number of research teams have proposed that VR reduces acute pain by drawing the patient’s attention into the virtual world, leaving less attention available for the brain to process incoming neural signals from the pain receptors. Consistent with this approach, in a quantitative sensory testing laboratory study of healthy volunteers using a within-subject repeated measures design with the treatment order randomized, Hoffman recently measured the effect of high tech vs. low tech VR on attention for the first time [37]. As predicted, VR analgesia was more effective during the high-tech vs. low-tech VR, and results on the divided attention task showed that high-tech VR also drained significantly more of the participants’ attention resources than low-tech VR, implicating an attention mechanism for how VR reduces acute pain [37].

Although subjective pain ratings are considered the gold standard of pain measurement, neuroimaging studies provide converging evidence to support the claim that VR reduces pain. fMRI brain scan studies have begun to explore changes in pain-related brain activity during VR vs. no VR during brief painful thermal pain stimuli at a safe, painful but tolerable temperature. In addition to reducing participants’ subjective experience of pain during VR vs. No VR, fMRI brain scans showed significant reductions in pain-related brain activity in all five brain regions of interest, (the ACC, primary somatosensory cortex, secondary somatosensory cortex, insula, and thalamus) [38].

In the current study, the significantly higher levels of fun reported by patients who used VR during venipuncture suggest that VR distraction can promote positive emotions and can help patients cope with the painful procedure. Some researchers speculate that VR reduces pain perception not only through a cognitive modulation (acting on the attentional resources), but also through an emotional activation (eliciting pleasure emotions). Hoffman, Sharar, and Coda et al., [34] introduced a new measure of “fun” during VR vs. during No VR as a surrogate measure of positive emotion. For example, in a clinical VR analgesia study that measured fun, patients reported that burn wound care during No VR was either no fun at all or mildly fun, whereas the same patients rated wound care during VR as “pretty fun” [39]. Gold, Belmont, and Thomas [40] pointed out that pain and pleasure share some neuropathways. Gold et al. speculated that in addition to an attentional mechanism, an affective mechanism may contribute to VR analgesia [39]. Similarly, Sharar et al. [41] speculated that providing a VR experience that maximally enhances positive affect (i.e., a fun VR world) could increase the analgesic effectiveness of VR. To date, several “low tech vs. high tech” laboratory studies have shown that increasing the immersiveness of the VR system makes VR significantly more effective at reducing pain and also makes VR more fun [37,42,43].

### 4.2. Limitations

The current study has some limitations: fear was not measured, and pain was only assessed during a single venipuncture, an important limitation. As mentioned earlier, children with chronic diseases typically have numerous venipunctures during their treatments. Whether VR continues to be effective during successive venipunctures per patient is an important research question. Previous studies with pediatric burn patients during wound cleaning suggest that VR continues to be effective when used during several treatments per patient [44,45,46]. All patients in the current study had previously received at least one venipuncture prior to participating. Future studies should report the number of previous venipunctures patients had received before participating. However, the results of Piskorz and Crub [30] were unaffected by the number of previous blood draws the patient had previously undergone. Another limitation is as follows. Although the Verbal Numeric Rating Scales used in the current study have been validated for use in children aged 6 and higher [31], it is possible that some children under the age of 8 may have difficulty in differentiating the components (e.g., pain unpleasantness, the emotional component of pain, and time spent thinking about pain during the procedure, the cognitive component of pain). The current study included five 7 year olds in a total sample of 82 patients. To address this possible limitation, an exploratory analysis of the results excluding the 7 year olds (not shown) also found no change in the conclusions.

Although the VR system used in the current study is relatively high tech compared with the earlier studies [19], much more immersive (e.g., wider field of view, higher resolution/crisper images, faster smoother update rate) VR goggles have recently become widely commercially available [37,47,48,49,50,51]. Several laboratory studies have shown that increasing the immersiveness of the VR system increases VR analgesia. For example, increasing the field of view of VR goggles significantly increased analgesic effectiveness [43], adding interactive eye-tracking increased VR analgesia [42], and adding interactive avatars significantly increased VR analgesia [37]. Moreover, in the future, multiplayer systems may be used to allow parents to “be there” in a shared virtual world with the patient to help comfort and distract the patient during the procedure. In light of recent technological advances, we predict that even larger clinical results will be obtained in future studies using the latest generation of more immersive VR systems. As a result of multi-billion dollar investments, VR is currently undergoing a dramatic series of breakthroughs, improving quality, greatly reducing cost and size, and making VR increasingly advanced but increasingly inexpensive and simple for novice users to operate. These developments greatly increase the feasibility of widespread dissemination of VR analgesia. VR has great potential to help reduce pain, reduce fear, reduce anticipatory anxiety, and to make venipuncture more fun. Augmented reality can also computer enhance the visibility of veins, reducing the number of venipuncture misses [52]. Ideally, when patients think about their experience days, weeks, or months later, they will remember pleasant experiences with the VR instead of unpleasant experiences with the needles (an interesting topic for future research). As scientists unlock the rich health information contained in blood samples, venipunctures are emerging as one of the most common procedures in medicine (especially for sick children undergoing treatments, to monitor progress and adjust treatments). Unfortunately, most children are afraid of needles and want to avoid venipuncture [7,8]. Avoidance of a simple venipuncture procedure can prevent potentially life-saving medical care, particularly vaccinations and early disease detection. VR distraction has shown efficacy for the temporary management of pain, fear, and distress associated with venipuncture using VR distraction (e.g., SnowWorld) in a range of populations [53,54]. The current results help establish that VR reduces pain during venipunctures in pediatric CKD patients, adding CDK to a growing list of patient populations that show VR analgesia during venipunctures.

## 5. Conclusions

This study contributes to a small but growing literature that supports the use of immersive virtual reality (VR) distraction as a psychological technique for pain control during venipuncture in patients with chronic kidney disease. VR significantly reduced worst pain, pain unpleasantness, and significantly increased fun during venipuncture. The patients in the current study also reported a strong illusion of going inside the computer-generated world during VR (7 on a 0 to 10 scale). The current study also provides the first exploratory post-hoc analyses showing a 55% reduction in worst pain during VR in patients aged 7–11 vs. a 35% reduction in worst pain during VR in patients aged 12–17, inconclusively raising the question of whether VR is more effective at reducing the pain of younger children vs. teenaged children, an interesting topic for future research.

Beyond distraction, future studies may explore new ways of improving well-being and health support in children affected by chronic diseases, e.g., breathing exercises, VR mindfulness coping skills [55,56,57,58], VR exposure therapy [59], and psychological treatments for chronic pain [18,50] for age-appropriate children/adolescents. Additional research and development is recommended.

## Figures and Tables

**Table 1:** Sample’s demographics and clinical features.

		No VR Group (N = 41)	Yes VR Group (N = 41)	Tot (N = 82)	*p* Value	Chi-Square
Age (mean ± SD)		11.39 ± 2.737	12.17 ± 2.626	11.78 ± 2.694	0.191	
Sex	Male	21	23	44		0.658
	Female	20	18	38		
Origins	Italy	35	37	72		0.390
	Albania	1	2	3		
	Philippines	2	0	2		
	Moldova	1	0	1		
	Senegal	1	0	1		
	Tunisia	0	1	1		
	Egypt	0	1	1		
	Ecuador	1	0	1		
Disease	Chronic renal failure	6	5	11		0.455
	Nephrotic Syndrome	5	5	10		
	Microhematuria	5	4	9		
	Proteinuria	4	2	6		
	Renal Transplantation	3	0	3		
	Gitelman’s Syndrome	2	1	3		
	Polycystic Kidney	0	3	3		
	Pseudohypoparathyroidism (PSH)	3	0	3		
	Glomerulonephritis	2	1	3		
	Renal Calculosis	0	3	3		
	Others (not specified)	11	17	28		

**Table 2:** (Ages 7–17). Pain, fun, and nausea mean levels in the experimental and control group.

	No VR Group Mean (SD)	Yes VR Group Mean (SD)	*t* Values	*p* (Sig 1-Tailed)
Worst pain	2.74 (2.76) 43% reduction	1.56 (1.83)	2.29	< 0.05
Fun	4.06 (3.74) 50% increase	8.06 (1.85)	−6.14	<0.001
Unpleasantness	2.41 (2.94) 52% reduction	1.17 (1.80)	2.31	<0.05
Time spent thinking about pain	2.78 (3.10) 11% reduction	2.48 (2.91)	0.46	>0.05 NS
Nausea	0.83 (2.16)	0.35 (0.99)	1.28	>0.05 NS

**Table 3:** (Ages 7–11). Pain, fun, and nausea mean levels in the experimental and control group.

	No VR Group Mean (SD)	Yes VR Group Mean (SD)	*t* Values	*p* (Sig 1-Tailed)
Worst pain	2.85 (2.93) 55% reduction	1.27 (1.75)	1.86	<0.05
Fun	5.55 (3.95) 36% increase)	8.60 (2.38)	2.64	<0.01
Unpleasantness	2.63 (3.45) 39% reduction	1.60 (2.41)	.98	>0.05 NS
Time spent thinking about pain	2.95 (2.98) 15% reduction	2.50 (3.25)	0.43	>0.05 NS
Nausea	0.90 (2.38)	0.63 (0.97)	0.41	>0.05 NS

**Table 4:** (Ages 12–17). Pain, fun, and nausea mean levels in the experimental and control group.

	No VR Group Mean (SD)	Yes VR Group Mean (SD)	*t* Values	*p* (Sig 1-Tailed)
Worst pain	2.64 (2.67) 35% reduction	1.73 (1.88)	1.37	>0.05 NS
Fun	2.64 (2.97) 65% increase	7.6 (1.70})	7.18	<0.001
Unpleasantness	2.21 (2.44) 58% reduction	0.92 (1.32)	2.31	<0.05
Time spent thinking about pain	2.62 (3.28) 6% reduction	2.46 (2.76)	0.48	>0.05 NS
Nausea	0.76 (1.97)	0.19 (0.98)	1.29	>0.05 NS

## Data Availability

The data presented in this study are available on request from the corresponding author. The data are not publicly available due to privacy policy of the hospital regarding minors’ data.
